# Implementing wastewater surveillance for SARS‐CoV‐2 on a university campus: Lessons learned

**DOI:** 10.1002/wer.10807

**Published:** 2022-11-13

**Authors:** Brian A. Wartell, Camila Proano, Lena Bakalian, Devrim Kaya, Kristen Croft, Michael McCreary, Naomi Lichtenstein, Victoria Miske, Patricia Arcellana, Jessica Boyer, Isabelle Van Benschoten, Marya Anderson, Andrea Crabb, Susan Gilson, Anthony Gourley, Tim Wheeler, Brian Trest, Glynnis Bowman, Birthe V. Kjellerup

**Affiliations:** ^1^ Department of Civil and Environmental Engineering University of Maryland College Park College Park Maryland USA; ^2^ Department of Residential Facilities University of Maryland College Park College Park Maryland USA; ^3^ Facilities Management University of Maryland College Park College Park Maryland USA

**Keywords:** campus, COVID‐19, SARS‐CoV‐2, wastewater surveillance, wastewater‐based epidemiology (WBE)

## Abstract

Wastewater surveillance, also known as wastewater‐based epidemiology (WBE), has been successfully used to detect SARS‐CoV‐2 and other viruses in sewage in many locations in the United States and globally. This includes implementation of the surveillance on college and university campuses. A two‐phase study was conducted during the 2020–2021 academic year to test the feasibility of a WBE system on campus and to supplement the clinical COVID‐19 testing performed for the student, staff, and faculty body. The primary objective during the Fall 2020 semester was to monitor a large portion of the on‐campus population and to obtain an understanding of the spreading of the SARS‐CoV‐2 virus. The Spring 2021 objective was focused on selected residence halls and groups of residents on campus, as this was more efficient and relevant for an effective follow‐up response. Logistical problems and planning oversights initially occurred but were corrected with improved communication and experience. Many lessons were learned, including effective mapping, site planning, communication, personnel organization, and equipment management, and obtained along the way, thereby paving an opportune guide for future planning efforts.

**Practitioner Points:**

- WBE was successful in the detection of many SARS‐CoV‐2 variants incl. Alpha, Beta, Gamma, Delta, Lambda, Mu, and Omicron.
- Careful planning and contingencies were essential for a successful implementation of a SARS‐CoV‐2 monitoring program.
- A surveillance program may be important for detection and monitoring of other public health relevant targets in wastewater incl. bacteria, viruses, fungi and viruses.
- Diverse lessons were learned incl. effective mapping, site planning, communication, personnel organization, and equipment management, thereby providing a guide for future planning efforts.

## Graphical abstract

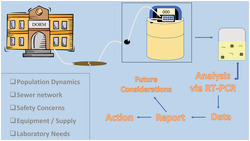

## INTRODUCTION

Wastewater surveillance, also known as wastewater‐based epidemiology (WBE), can help predict and monitor microorganisms in wastewater such as polio‐ and coronaviruses (Jafferali et al., 2020; Michael‐Kordatou et al., 2020). Yet, although coronaviruses are primarily airborne, their RNA can remain intact in stool samples (Twigg & Wenk, 2022). Thus, monitoring of viral RNA through sewage can effectively be used to determine the presence and abundance of the pathogen within a community or, in this case, a dormitory or group of buildings at a university campus (Larsen & Wigginton, 2020; Peccia et al., 2020). Coronaviruses, such as SARS‐CoV‐2, can be easily detected in human feces and thereby also in wastewater (Saawarn & Hait, 2020; Sbaoui et al., 2021). SARS‐CoV‐2 has been detected in wastewater through many studies and has been heavily researched since the early days of the COVID‐19 pandemic in early 2020 (Larsen & Wigginton, 2020). Furthermore, as SARS‐CoV‐2 is more infectious and transmissible than other coronaviruses of concern (e.g., SARS‐CoV‐1 and MERS‐CoV), it is imperative to control its spread (Maal‐Bared et al., 2021). WBE is an important public health measure, as it is unbiased and includes in its detection those individuals who are either asymptomatic or do not report to a health facility (Barua et al., 2022; Mao et al., 2020; Polo et al., 2020). Furthermore, it is less likely that these individuals will be screened or otherwise detected. Therefore, WBE has an advantage as it includes all of these individuals indiscriminately (Gao et al., 2021; Hewitt et al., 2022; Patel et al., 2021).

Numerous colleges and universities, therefore, have sought to implement wastewater surveillances (WBE) on their campuses. This was especially true during the height of the initial pandemic, namely, the 2020–2021 academic year. In September 2020, United States news outlets published about 200 articles on wastewater surveillance efforts for SARS‐CoV‐2 on college campuses (Harris‐Lovett et al., 2021). As of January 2021, more than 210 colleges around the world had initiated steps to begin wastewater monitoring, and many more were considering following suit (Harris‐Lovett et al., 2021). This number increased to 248 college campuses as of April 26, 2021 (University of California Merced, 2021), near the end of the Spring 2021 semester.

Below are brief summaries of a few selected studies that were published at or shortly following the time at which this paper's study concluded. These particular studies were chosen either because of their success in implementation or to include a variety of diverse locations and student population sizes.

The University of Arizona (UA) and the University of North Carolina at Charlotte (UNC) are both large urban universities, having similarly integrated WBE programs on campus. UA successfully utilized WBE in conjunction with clinical testing to obtain a representative collection of COVID‐19 cases on campus, delivering an 82.0% positive predictive value and an 88.9% negative predictive value. These efforts helped to prevent potential transmission from at least three infected students (Betancourt et al., 2021). Unlike the UA, the UNC has much of its student population living in on‐campus dorms and thus emulates a similar characteristic to the University of Maryland. UNC's use of WBE identified multiple asymptomatic individuals who had not been discovered through other parts of the COVID‐19 campus monitoring program (Gibas et al., 2021), again highlighting the added benefit of utilizing WBE. The University of California, San Diego (UCSD), also, has an urban to a more unique approach to WBE. As part of its robust Return to Learn program, the UCSD instituted a comprehensive wastewater sampling initiative (via robotic‐driven analysis and autosamplers) to help reduce the spread of COVID‐19 on campus (UC San Diego, 2021). An efficient coordination of WBE with other testing and health initiatives, combined with a relatively small student body (10,000 students), managed to keep COVID‐19 case rates far lower than much of the surrounding community (UC San Diego, 2021). Perhaps the most efficient use of WBE was seen at Hope College (HC), a small suburban college in Holland. Primarily because of its small size, HC was able to cover approximately 55% of its entire student population, including 70% of campus‐owned housing. The WBE was integrated with the clinical findings to create a more comprehensive approach, helping to identify and isolate many asymptomatic cases (Travis et al., 2021).

In an effort to establish similar success, a WBE effort was put in place at the University of Maryland. The initial goal, taking place during the Fall of 2020 (Period 1), was to create a pilot study that would encompass the majority of the on‐campus student body and compare findings to clinical tests on campus. This would complement additional studies being performed such as surface tracing and air sampling. The primary objective overall was to determine if wastewater surveillance would be possible at such a large university, being able to coordinate with many different administrative and other departments. The goal of Period 2, which took place in the Spring of 2021, was to establish more refined parameters, targeting a smaller pool of on‐campus students but with greater accuracy.

In the Fall of 2020, the University of Maryland began to frequently test individuals for the SARS‐CoV‐2 virus or COVID‐19 symptoms, mandating daily reporting of symptoms or contact when present on campus. Clinical testing was initially required monthly but was suggested more frequently. Infected individuals were removed and placed in isolation and/or quarantine housing. However, a significant number of COVID‐19 cases still occurred throughout campus, presenting a need for faster and cheaper detection. WBE was chosen as a potential aid as it is able to detect the virus in a given group of individuals up to 3–5 days before symptoms present themselves. It is therefore useful in detecting presymptomatic and asymptomatic individuals (Michael‐Kordatou et al., 2020). Nasal swabs or similar clinical testing, although capable of detecting asymptomatic individuals, often miss those individuals who do not report or notice symptoms (Bibby et al., 2021). This makes WBE an important source of data within a community that can be used in combination with clinical testing (Michael‐Kordatou et al., 2020).

The goal of this effort was to establish a framework for the detection of COVID‐19 clusters on campus in a pilot study, with the overall objective of preventing the spread of the virus within the community. “Clusters” are defined as three positives based on a specific location, proximity, and contact tracing. If five positives occurred based on these criteria, it would be considered an “outbreak.” Fortunately, there were few “outbreaks,” but they did occur. The Fall 2020 semester's tactic therefore was to monitor as much of the on‐campus population as possible to obtain a comprehensive understanding of where the virus was spreading. The second semester's tactic, however, shifted to target smaller groups of residents on campus in order to establish an effective follow‐up response. In both instances, a collaboration of team members from different departments (see Section 3.1) was successfully utilized, highlighting the benefit of coordination between many different roles of university faculty.

A key lesson learned was selecting strategic testing sites that allow for a representative sample while being logistically accessible. Other key lessons were being able to anticipate and problem‐solve for weather challenges and keeping open communication with all parties involved in the project. Based on the findings of this initiative, the infrastructure is largely in place to perform wastewater monitoring for a campus‐scale project and can be used for other types of monitoring besides SARS‐CoV‐2. The publication of this study is beneficial as it seeks to highlight the challenge, obstacles, and adaptations needed to implement a WBE program on a large semiurban campus.

## METHODS AND MATERIALS

This project can be separated into two different time periods during which the methods differed slightly. Period 1 encompasses the Fall 2020 semester (September–December 2020), where proof of concept testing was conducted. Period 2 includes the start of the Spring 2021 semester (January–March 2021), where a pilot program ran for approximately 6 weeks. Specific differences between these two time periods are further explained in each section of the methods and materials.

### Location selection

During Period 1, six individual sites were selected and subdivided into two sections. These two sections corresponded to residence hall clusters on the north and south geographical locations of the campus community. The original goal for Period 1 was to survey all of the occupied residence halls across campus; therefore, the sample locations were chosen in a manner that all halls could be sampled with six sample sites. The vast majority of occupied housing buildings were included in this study, with the exception of one hall grouping and Greek housing. Limitations and exclusions resulted from limited autosampler availability at that particular time.

The site selection for Period 2 was heavily modified after reconsideration of the goal to focus on individual halls and specifically a much smaller population size (<200 students). With a smaller population size, a more tailored and targeted transmission mitigation approach could be taken by the public health officials on campus. The large sample size (500–1000 students) utilized during Period 1 made it difficult to take precautionary or reactionary measures to the findings in the wastewater.

Locations chosen for Period 2 were initially based on specific hall locations with a greater number of reported COVID‐19 cases (via nasopharyngeal swabs, saliva tests, and contact tracing). Site choices were later refined based on three main factors: feasibility of access, sampling logistics, and ability to collect samples. With regard to feasibility of access, some sites were preferred over others because of their accessible infrastructure. Therefore, dedicated sanitary manholes were deemed more preferable. Furthermore, some manhole locations were located in streets or other central walkways, thus making them inaccessible for long‐term monitoring. Sites were thus chosen where they were not impeded by the flow of foot traffic and would not disrupt daily community activities.

Additionally, sampling logistics further narrowed down the selected sewer manholes. The flow in some sewers combined wastewater with greywater, making them less preferable sampling locations. Additionally, having fewer students on campus (roughly 50% occupancy) led to lower than anticipated sewer flows, thus creating periodic difficulty in sample collection and the need to implement low‐flow strainers at most locations. Other sampling logistics included considerations for the population that was intended to be sampled. Some of the sanitary sewer lines drew from bathrooms on a particular floor or wing of a dormitory. In these cases, and when communal hallway bathrooms were sampled, our results had to be evaluated for the sample of the population they were drawing from (i.e., one floor of a five‐story building, one dedicated hallway, or all of the toilets in the building). Furthermore, this also led to discussions of the uncertainty, as it was not possible to control or estimate which restroom facility is used by a specific resident. This uncertainty contributes to the reactionary measures that could be taken from positive cases in the wastewater and will be further discussed in Section 3.1.

The ability to collect samples is affected by the documentation of a historic campus, as some building plans were different than expected or were not applicable to the goals of this project. Buildings used by the general public and not a consistent set of residents such as a dining hall, recreational facility, or a student activities building are not used by the same set of residents every day, making it difficult to correlate a positive sample result to a specific subsect of the population.

On occasion, a site had to be relocated, and this is explained in Section 3.1.

### Autosamplers details

**FIGURE 1:**
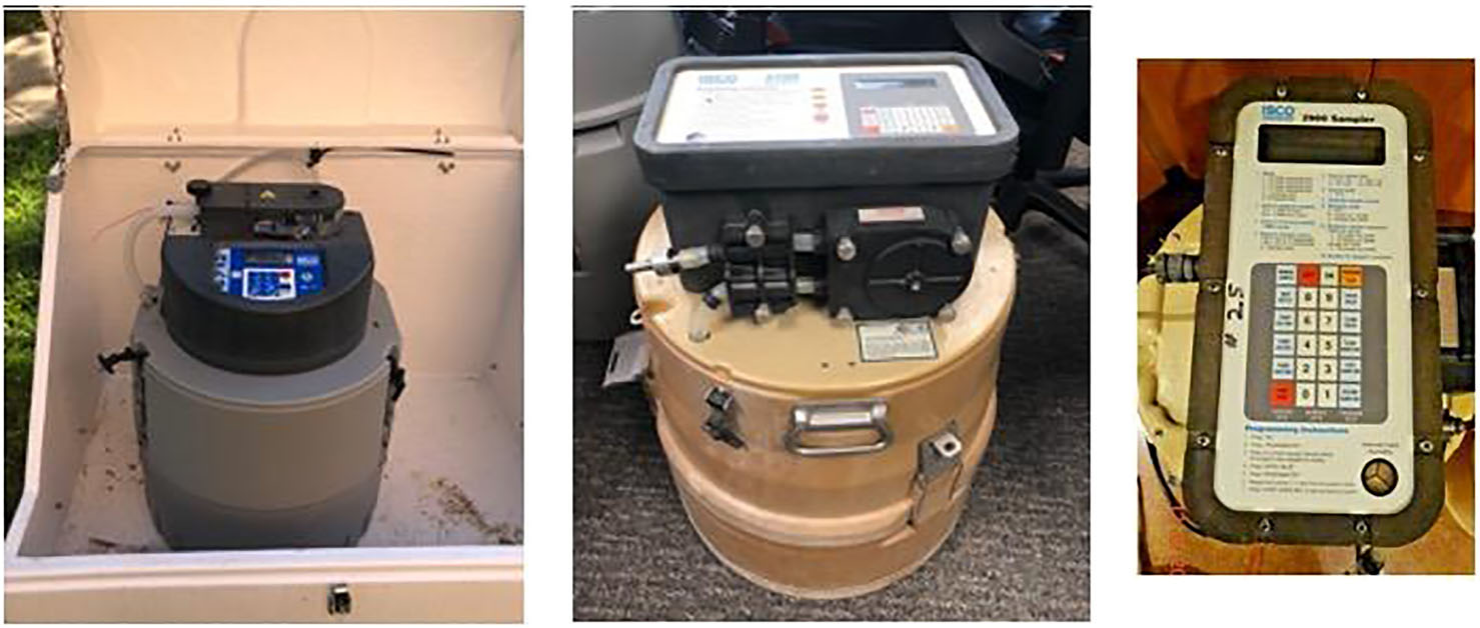
Left: ISCO 6712 autosampler as installed in the field. Center: ISCO 3700 being prepped for usage. Right: ISCO 2900 autosampler panel (closeup)

Three autosampler models were utilized for this project: ISCO 2900, ISCO 3700, and ISCO 6712 (Teledyne, Lincoln, NE, USA) (Figure 1). During the Fall 2020 semester, only models 2900 and 3700 were utilized. Newer autosamplers were unable to arrive until the end of November 2020, shortly before the end of the Fall 2020 semester. For the Spring 2021 semester, as the new models had arrived, 6712 models were utilized along with some of the 3700 models. All models served the same purpose and were programmed in identical manners. The only difference between models was the user interphase and the appearance of the samplers (Figure 1).

**FIGURE 2:**
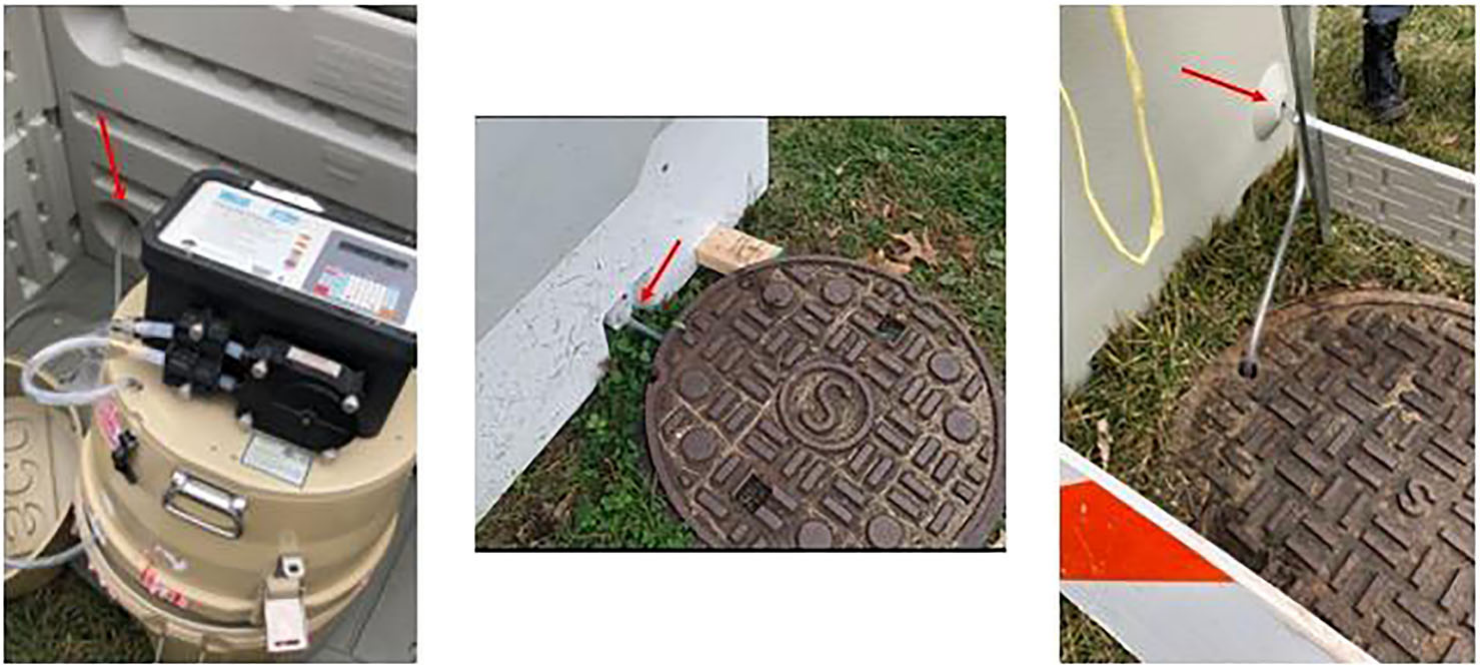
Left: Tubing extending outward through the rear of the security box; Center: Tubing coming up the sewer into the security box from beneath manhole cover; Right: Tubing extending directly into manhole opening (one location only)

The autosamplers functioned by drawing wastewater from the sewer into a 5‐gal collection jug inside of the sampler. A sample strainer was attached to vinyl tubing and placed into the sewer line. The tubing designed for the wastewater sampling (Teledyne, Lincoln, NE, USA) was measured and extended upward above ground to each autosampler (Figure 2). Each autosampler had a different pump rate depending on the distance from the ground to the bottom of the sewer line, as the depth varied between 3 and 25 ft for Period 1 locations and 12–25 ft for Period 2 locations, respectively. The pumping rate was determined by the software of the autosamplers.

Each sampling unit was placed inside a security box (Storm Box™, Precision Systems, Calumet City, IL, USA) to keep it safe from tampering as well as to provide protection from harsh weather conditions that would affect the performance of the sampler. During the warmer months, ice blankets were wrapped around the collection jugs to preserve the samples.

Most samples in Period 2 and some samples in Period 1 were designed to be composite samples. The composite samples were highly preferable, as they provided a more representative sample of SARS‐CoV‐2 levels over the particular time period, as they would include a larger percentage of the studied population and were scheduled to collect during peak flow hours. Composite samples relied on the autosampler programming and collected samples periodically (see Section 3.3). When initiated, the device purged the tubing suction line for a few seconds and then began to pump forward until the specified volume was collected. Once the desired volume had been collected, the programmed device purged the line to rid it of any leftover wastewater residing in the tubing. This was done to avoid dilution or contamination of future‐drawn samples as well as to reduce the possibility of frozen wastewater in the line during the winter months.

There were instances when a machine grab sample was necessary, such as initial setup prior to the composite collection, or complications that prevented samples from being aggregated during the allotted time period (Section 4.3). This sampling technique was reserved for instances where composite sample collection failed. Failure to collect could be attributed to clogs in the line, dying batteries, or low pressure in the sewer (see Sections 4.2 and 4.3). To obtain a machine grab sample, the autosampling device could be manually operated to pump forward or to take a programmed grab sample. With the former function, it was possible to retrieve any amount of sample desired, as the program would pump until the stop button was pressed.

### Wastewater collection

Wastewater (WW) samples were often collected twice a week, primarily as composite samples. During Period 1, 90–200 ml of WW liquid per half‐hour was collected over a 48‐ to 96‐h duration. In Period 2, the autosamplers were programmed to collect 130 ml samples initially over a 48‐ to 72‐h period and then reduced to a 24‐ to 30‐h period. In both Periods 1 and 2, samples were most often collected on Monday and Thursday mornings, weather permitting. During part of the winter, there were difficulties in collecting samples because of weather‐related phenomena. In these cases, sampling schedules had to be shifted, and samples were a combination of composite and grab samples.

For sample retrieval, a team of two people would visit each collection site and fill up labeled HDPE sample bottles, which were transported back to the lab in a cooler filled with ice or ice blankets. Eye and body safety was implemented as per University protocol. To prevent any pathogens from being inside the cooler or in the transportation vehicle before bottles were placed in the cooler, the outside of each sample bottle, and any soiled equipment, was sanitized with ethanol (70% v/v). For ease of use, a plastic caddy was used to carry all of the necessary materials for collection. In this caddy, the sample bottles, storage box keys, sanitizer, extra gloves, and paper towels could be stored and carried.

Upon arriving at each site, the large collection jug was inspected to determine it had collected sufficient WW sample volume for analysis (≥300 ml). On average, the container was roughly filled halfway (~10 L). Next, the collection jug was removed from within the autosampler and lightly shaken to disturb the solid formation and create a more homogenous composite sample. A smaller sample was then taken from this collection jug (either 500‐ or 1000‐ml HDPE sample bottles were used). During Period 1, both sizes were used indiscriminately. During Period 2, 500‐ml sample bottles were reserved for composite samples, whereas 1000‐ml bottles were used for grab samples. On average, the sample bottles were filled to 50%–85% of their capacity.

Once a sample bottle was filled, the remaining liquid in the collection jug was carefully poured back into the sewer with the aid of a plastic funnel. In all locations save for one site, the manhole cover was left slightly propped open to allow for this liquid waste to enter, as well as to not pinch the tubing inside it. Any wastewater spills were sprayed with the ethanol mixture and wiped with paper towels, as necessary. Clean tap water was used to rinse the collection jug, allowing any solids left in the jug to be washed away. Once the collection jug was rinsed, it was returned to the inside of the autosampler with the hose placed inside, the sampler was programmed to start the next collection time.

Batteries were replaced once a week, or as needed. Cold temperatures and the new autosampler model (6712) would drain the battery faster, and thus in some cases, it was necessary to replace the battery twice a week. Once at the lab, the bottles were shaken and mixed thoroughly to provide a representative sample. The samples with a higher solids content were shaken more vigorously to disturb any solids that may have settled to the bottom.

### Analytical procedures

From each sample, postcollection (≤1000 ml), 90 ml of wastewater was taken and divided into two 45‐ml aliquots. These aliquots were then concentrated to approximately 1 ml and processed via centrifugation, ultrafiltration, and concentration. Specifically, each 45‐ml aliquot, in 50‐ml Falcon® conical tubes, was centrifuged in a benchtop centrifuge (Allegra X‐22, Beckman‐Coulter, Brea, CA, USA) at a speed of 3400 G for 20 min to remove the solid fraction and large particles. The resulting supernatant was then transferred to new centrifuge tubes and apportioned over multiple centrifugal cycles into 15‐ml Amicon Ultra‐15 Centrifugal Filter Devices (100 kDa cut‐off) (Millipore, Amsterdam, the Netherlands) and then centrifuged for 15 min at 3400 G. This yielded a concentrated residual liquid of 1–1.5 ml, with duplicate samples (45 ml) combined. After concentration, samples were transferred to sterile 1.5‐ml tubes (Invitrogen, Carlsbad, CA, USA) for subsequent RNA extraction. RNA was extracted via the Zymo Quick RNA mini prep R1055 kit (Zymo Research, Irvine, CA, USA) and was converted to cDNA according to the methods outlined in the NEB #M0368 Standard Protocol (New England Biolabs, 2020). It was then processed and analyzed by RT‐qPCR via the CFX Connect Real‐Time PCR Detection System (Bio‐Rad, Hercules, CA, USA), using standard and calibration curves to determine the viral concentration.

Protocols used were based on CDC recommendations, as outlined by Corman et al. (2020) and Lu et al. (2020). Plasmids containing the SARS‐CoV‐2 gene (N) provided with the 2019‐nCoV RUO Kit (Cat. No:10006625, IDT) were linearized by following the manufacturer's (IDT) protocol prior to their use as an RT‐qPCR standard. Standard curves for CDC N1 assays were prepared as serial dilutions (10‐fold) in order to determine quantitative results. Modifications and specifications used are outlined in Kaya et al. (2022). De‐ionized water was processed along with the collected WW samples as a field and extraction blank. RNAse‐free water was used (separately) as method blanks and negative controls.

Additionally, prior to processing, samples were spiked with Bovine Respiratory Syncytial Virus (BRSV) (Inforce 3 Cattle Vaccine™, Zoetis, Parsippany, NJ, USA) as a surrogate for quality control of the procedures (BRSV was added at a ratio of 1 μL per 1 ml of sample). Specifically, for each 45‐ml aliquot of the sample, BRSV was added (at a 1:1000 ratio) from stock concentration with a median concentration of 10^11^ ± 10^1^ gc/L. The reported recovery was approximately 8.5%, and values typically ranged between 0.5% and 20%, consistent with results in similar studies (Alygizakis et al., 2020; Bivins et al., 2021; Prado et al., 2021).

## DECISION MAKING

### Site selection

Site selection is an integral part of this process in ensuring a representative sample can be feasibly drawn. Although the initial sites chosen (Period 1) were placed at locations to sample from the majority of occupied housing, these locations changed throughout the span of the project to facilitate an actionable response time. Specifically, during Period 1, the goal was to procure a sample from (nearly) every residence on campus, which resulted in sites collecting samples from large hall “clusters,” or groups of multiple buildings. Through this tactic, six residence housing clusters out of the seven identified were able to be monitored (Table 1). However, it became clear that this was not practical in order to elicit a proper reactionary response by the university, as it encompassed too large of an area to pinpoint exactly where the virus was detected from (see Table 1). This tactic shifted during Period 2; sites were placed to obtain samples from no more than 200 students each, compared with as many as 2195 from Period 1. These sites were selected based on where a higher number of positive cases had historically been reported on campus. This shift in strategy allowed for a better chance to pinpoint the emergence of the virus within a particular dorm, at times, even to a particular floor or wing of the building. This allowed for more effective use of wastewater monitoring as it allowed for the University Health Center to take action if they chose to do so.

**TABLE 1:** Sampled sites and their respective dorms for the Fall 2020 and Spring 2021 semesters

Fall 2020			Spring 2021		
Location	# of initial students	# of dorms	Location	# of initial students	# of dorms
Site “A”—South 1	~225^a^	4	Site “A”—South 1A	200–250	2
Site “B”—South 2	~1000	9	Site “B”—South 1B	~110	1
Site “C”—South 3	~525	16 (low occupancy)	Site “C”—South 4	~100	1
Site “D”—North 1	~1000^b^	4 (high occupancy)	Site “D”—North 1	100–1000^c^	4
Site “E”—North 2	~700	3 (high occupancy)	Site “E”—North 4	~130	1
Site “F”—North 3	~650	5	Site “F”—North 5	~130	1
			Site “G”	~140	1
			Site “H”	~140	1
			Site “I”	~45	1

*Note*: Distances of all sites were within 1 mile of the campus laboratory.

^a^ Number provided for both dorms and suites.

^b^ Number provided for entire estimated input from dorm community.

^c^ Was planned to be more isolated but had combined input from multiple other dorms.

Additionally, some initially chosen locations had to be abandoned and relocated, because of the inability and inconsistency of sample collection. One of the selected locations was relocated a couple of weeks into Period 2 because of inconsistent water flow, leading to intermittent sample collection. The autosampler at this location was not able to always draw water from the sewer channel, even when low‐flow strainers were used (for more information on low‐flow strainers and their use please refer to the lessons learned section). Although the autosampler was sometimes able to take grab samples, the variation in waterflow contributed to failed composite sampling. It was confirmed that the residence buildings should have a significant number of residents, but as capacity was lower than typical, it may have reduced the expected flow. If the flow was amply sufficient, it may have been that the design of the sewer channel hindered the collection of composite samples.

The logistics of placing the autosamplers around the campus heavily involved the Facilities Management in understanding the pipe layouts, how pipes connected to other waterways, and in the way each waterway was constructed. Clear communication was key for needing manholes opened, maintenance, and other logistics. This process was a highly collaborative one with the Facilities Management as well as with other departments, such as Administration, Health Center, Geology department, and the Department of Residential Facilities. At times, planned locations were discovered to not be accessible, or were commingled with wastewater from other dorms. There also were a couple instances where the sewer line map was not entirely accurate, although this was a rare occurrence. This is something that certainly should be considered, particularly at older campuses with complex infrastructure.

Although the University of Maryland campus does not have a combined sewer system, this would be important to note in future initiatives for campuses that do have a combined sewer system; this would greatly dilute the samples drawn and might not be a viable source to detect RNA from. It would also be important to note how much water comes from greywater such as showering, as this may dilute the sample as well as provide potentially inaccurate results. A surrogate such as the Pepper Mild Mottle Virus (PMMoV) can be utilized for this purpose to normalize the WW sample for the relative solids content.

### Tubing selection et al.

The tubing of the autosamplers caused various sampling complications. Initially, the tubing and fittings used were what came with the borrowed autosamplers. With the exception of the inner machine tubing, most was not the proper tubing recommended by the manufacturer. Some of the tubing was too rigid for the motor to turn efficiently, which resulted in no production of suction to draw the sample. To remedy this, a softer tubing was used where the tubing met the motor mechanism, and that tube was connected to the harder tubing to go into the sewers. During Period 2, new tubing was installed to address incorrect tubing issues and worn‐out parts. The two respective tubes were connected by pushing one into the other, which held them together, but the security of the connection could vary between tubes based on hardness and sizing. Fittings were used to connect sections of tubing together, which were excellent in making a secure connection and allowing them to easily be separated and joined together.

### Sample frequency

The timing of the sample collection by each autosampler could have affected the reliability of results. Since samples were collected only twice a week; this timing may not have accounted for potential positive results that occurred between those two times. Increasing this frequency could yield more accurate and current results but would require more personnel, working hours, and laboratory usage. An additional benefit, however, to collecting more frequent samples would be reducing the likelihood that a positive sample was missed. Rather than a sample being collected every 2 to 4 days, a more frequent sampling pattern could be ideal to help identify cases on campus, but this would weigh heavily upon personnel. Similarly, intervals between sampling draws could be shortened. For example, instead of drawing a sample every 30 min, a sample could be pulled every 15 or 10 min. This could produce a more representative sample that might yield more comprehensive results but also poses some practical issues, including having accumulated volume in the tank. However, based on findings and results, a shorter span between autosampler draws does not appear to be necessary as the viral RNA appeared to remain in the system and corresponded well with the findings of the health center. This was often true even with grab samples, although to a lesser extent. The overall summary of detections for all dorms tested can be seen in Tables 2 and 3. Values were determined as averages of samples (*n* = 3), and the detected values ranged from 5.5 × 10^4^–2.1 × 10^8^ gene copies per liter (gc/L). These values of SARS‐CoV‐2 are reported as extrapolated for the viral titer in the original WW sample. The lowest raw output RT‐qPCR value (LoQ) was approximately 1 × 10^5^ gc/L. Below this level, no signal was detected. These values were considered “negative.”

**TABLE 2:** Sampled sites and positive values determined for the Fall 2020 semester (Period 1)

Site	Location	17 Sep	23 Sep	1 Oct	7 Oct	14 Oct	19 Oct	21 Oct	26 Oct	29 Oct	2 Nov	5 Nov	9 Nov	13 Nov	16 Nov	19 Nov	24 Nov	1 Dec	3 Dec	7 Dec	10 Dec	15 Dec	17 Dec
A	South1																						
B	South2																						
C	South3																						
D	North1																						
E	North2																						
F	North3																						

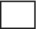
 = negative; 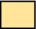 = borderline‐positive/inconclusive; (<1 × 10^5^ gc/L) 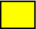 = moderately positive; (1 × 10^5^–1 × 10^6^ gc/L) 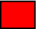 = strongly positive;(1 × 10^6^–5 × 10^7^ gc/L) 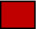 = very strong positive (>5 × 10^7^ gc/L).

*Note*: Samples were deemed positive if at least two of the three replicates had a detected value via RT‐qPCR. Strengths of positives were based on relative ranges of all positive values observed.

**TABLE 3:** Sampled sites and positive values determined for the Spring 2021 semester (Period 2)

Site	Location	28 Jan	3 Feb	8 Feb	10 Feb	15 Feb	17 Feb	22 Feb	25 Feb	1 Mar	5 Mar
A	South 1A										
B	South 1B										
C	South 4										
D	North 1										
E	North 4										
F	North 5										
G	Site G										
H	Site H										
I	Site I										

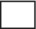
 = negative; 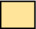 = borderline‐positive/inconclusive; (<1 × 10^5^ gc/L) 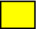 = moderately positive; (1 × 10^5^–1 × 10^6^ gc/L) 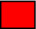 = strongly positive; (1 × 10^6^–5 × 10^7^ gc/L) 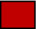 = very strong positive (>5 × 10^7^ gc/L).

*Note*: Samples were deemed positive if at least two of the three replicates had a detected value via RT‐qPCR. Strengths of positives were based on relative ranges of all positive values observed.

Detected (extrapolated to original WW) values below 5 × 10^5^ gc/L with only two positive replicates, and all values below 1 × 10^5^ were deemed “borderline‐positive.” Values between 5 × 10^5^ and 1 × 10^6^ and values between 1 × 10^5^ and 5 × 10^5^ with three positive replicates were deemed “moderately positive.” Values between 1 × 10^6^ and 5 × 10^7^ were deemed “strongly positive.” Values above 5 × 10^7^ were deemed as “very strong positives.” The decision to use these ranges was based on a combination of literature and the spread of different value magnitudes occurring on a specific date.

**FIGURE 3:**
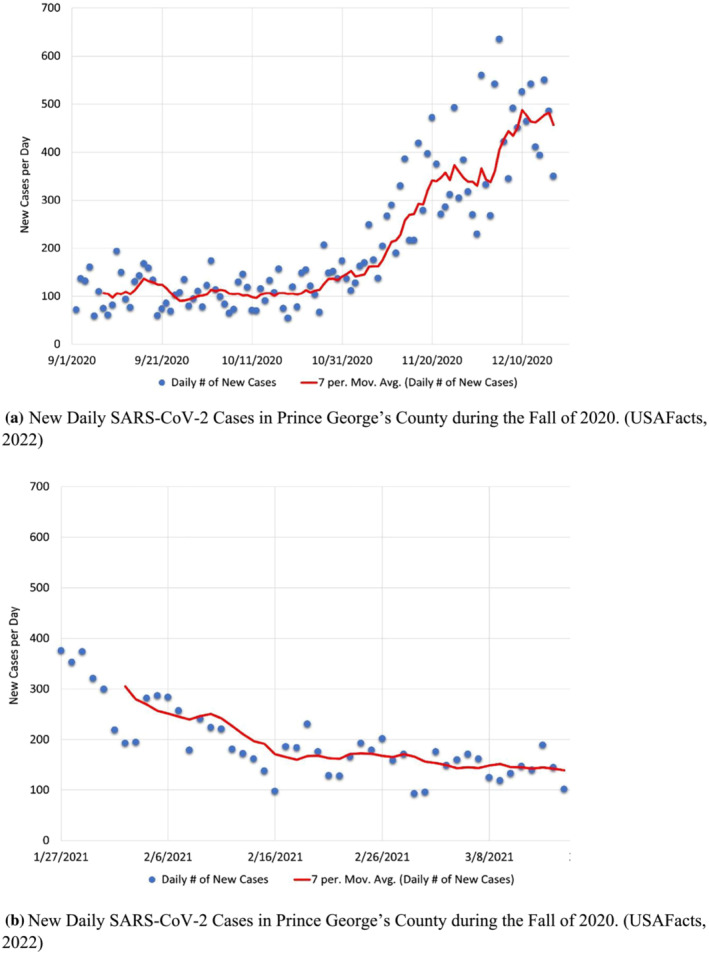
(a) New daily SARS‐CoV‐2 cases in Prince George's County during the Fall of 2020. (USAFacts, 2022). (b) New daily SARS‐CoV‐2 cases in Prince George's County during the Fall of 2020 (USAFacts, 2022)

The relative heat map of cases on campus shows both a discrepancy and a similarity with trends seen in the surrounding county (Prince George's County, MD). The initial surge in cases, as observed from September to early October was likely confined to the campus spurred on by new students moving in. However, the larger peaks in values, ranging from mid‐November to mid‐December, are consistent with a rising in cases on the county level, as is seen in Figure 3a.

It would be expected that the data obtained from campus during the Period 2 (Spring 2021) would be less representative of the county trends, as only select dorms were chosen and a much smaller number of students were sampled. This was generally found to be the case. However, the county data (Figure 3b) indicate a decreasing trend in cases until mid‐February. This, as a whole, aligns with the fewer positive cases detected on campus. Although on a county level, from mid‐February, the cases no longer decrease, they do not notably increase. However, on campus, at the specific locations sampled, there was a very large increase in both the number and magnitudes of positive cases detected. This may have been indicative of a spread isolated largely to the campus. This indication coincided with clinical testing results conducted by the University. Therefore, this helped to contribute to an overall awareness of viral spread and increased testing frequency and quarantine procedures. Indeed, similar trends have been noted in the literature where Betancourt et al. (2021) noticed increases specifically on campus after large holiday student gatherings and Wong et al. (2021) noted that spikes in cases can occur on a college campus even when the surrounding area has very low infection rates.

## SAMPLING AND RELATED OBSTACLES

### Timing

Period 1 presented many issues that accompanied the rollout of this project. For this reason, the time it took to complete sampling was highly variable. The sites were more spread out as compared with the second semester, so sampling generally took around 2 to 3 h to complete for the six sites. During Period 2, the samples were generally brought to the lab in around 2 to 3 h from the nine sites, depending on the number of problems faced and the weather. If possible, having multiple teams collecting samples would reduce the amount of time from when a sample was collected to when a sample was processed. More importantly, this would reduce stress on personnel and would allow an earlier processing time in the laboratory.

The overall process of collecting samples to obtaining results averaged 24 to 30 h to complete. The total sampling process for nine sites generally took about 3 to 4 h. Once the samples were brought back to the lab, the processing required approximately 6 to 8 h. The cDNA and qPCR steps were generally not completed on the same day, so the results were reported the following day. Personnel resources need to be sufficiently allocated to permit timely collection and analysis of samples and allow prompt action in the event of positive samples.

### Autosampler issues

The autosamplers presented some unforeseen technical difficulties, including dying batteries and a malfunctioning keypad. If the autosampler battery was low but the site had collected a successful composite sample, the battery was replaced for the next collection. If the battery had died prior to collecting a successful sample, a fully charged battery was installed to take a grab sample and the sampler was programmed to collect a composite sample for the next collection. Being able to clearly differentiate the old and new batteries was important; placing a piece of colored tape on the used battery could be a useful tactic in keeping track of the charged and empty batteries. Another idea could be to place the used batteries in a designated bin.

The newer autosamplers drained the battery about three to four times faster than the older models did. The colder temperatures also seemed to reduce battery life and resulted in more frequent battery changes. The issues with the batteries resulted in fewer successful composite samples and consequently, more grab samples. To attempt to address this, batteries were kept charging after each day of sampling so they could be replaced as needed on the next day of sample collection. The lab had a limited number of chargers (five), so it was not possible to charge the batteries for each site after every sampling. More batteries and chargers would have been ideal for more efficient sampling.

For one of the autosamplers during Period 1, part of the keypad did not function and led to more erratic sample volumes and draw times. However, although this impacted uniformity, it did not seem to impact any of the results. The autosampler was replaced during Period 2.

### Problems occurring while collecting samples

Obtaining a representative sample sometimes posed a challenge because of maintenance issues, such as clogged tubing. From mid‐December to mid‐January, fewer residents on campus due to the Winter Break resulted in a lack of flow in the sewers. This caused an accumulation of solids inside the tubing of some of the autosamplers that remained on campus during this time. To remediate this issue, the tubing was disconnected, and the debris was removed through a series of forward and reverse pumping. Once the tubing was cleared, it was reconnected to the system.

In future wastewater sampling initiatives, it is important to regularly clean sampling strainers and ensure that autosampler intake is not impacted by debris in the sewer. Initially, a larger plastic strainer was used but was then changed to a heavier metal strainer because of issues with it being too light to stay positioned correctly in the sewer. This was problematic in areas with low flow, so, where needed, these strainers were changed out for metal low‐flow strainers, which were smaller in size. Although this helped with the flow, these strainers were more prone to being clogged by sanitary items flushed down the drain (flushable wipes, dental floss, etc.) and other debris. Although both plastic and metal strainers were used for this project, neither was particularly effective at avoiding clogging issues. The metal low‐flow strainers were the best in most scenarios.

The colder months and freezing temperatures brought the additional issue of wastewater freezing in the tubing. When the wastewater froze in the tubing, it prevented regularly scheduled composite sampling from occurring because of blockage in the tubes as well as the tubes becoming too rigid because of the cold temperatures to allow flow. An attempt at remediating this issue was to place insulation around some of the accessible parts of the tubing, but this did not seem to make much of a difference in preventing frozen wastewater in the tubing. If a grab sample was taken, it was important to manually purge the tubing afterward by pumping in reverse. This reduced the chance of water staying in the tubes and having time to freeze. The autosampler was programmed to purge the tubing after each sample collection, which was important in keeping the tubing ice‐free.

When a grab sample was needed because of a lack of composite sample, the frozen parts needed to be flushed first. To do this, rinse water was reverse pumped through the tubing to dislodge the ice and then was pumped forward again to obtain a grab sample. Oftentimes, manual agitation of the tubing was necessary to clear out the tubing.

## FUTURE INITIATIVES

### What similar initiatives should consider

Researchers and stakeholders should collaborate early in the process to decide on the sampling strategy including who the target populations are, what potential results may be and actions to be taken when positive results are found. “Positive” should be clearly defined and is dependent upon the calculated threshold of PCR results and related scientific measurement, confirmation of replicates, and overall jurisdiction and decision‐making by campus health officials. It should also be more clearly determined when positive results are significant enough to elicit a response from campus or health officials.

Having a more accessible point of access to the manhole/sewer would have been beneficial in potentially reducing the amount of spillage around the area and in allowing for more accessibility for the user to perform this action. Useful items for successful sampling included a car‐seat tarp to protect the driver's vehicle from any potential spills or contamination and a trash bag for used paper towels and dirty gloves. A portable safety kit would be very useful to have in the car in the event that on‐site aid is needed.

A car was used to drive around campus and collect samples and allowed for storage of many materials and equipment. A large‐size car with ample trunk space would be preferable to a smaller‐size vehicle for storing equipment and samples. Additionally, a car that is able to drive partially off‐road would be useful in accessing sites that are not on or next to a paved roadway. This task would be difficult to accomplish on foot if the sample sites are at a considerable distance away from each other. A parking pass was used which allowed for sampling around a campus that has many parking restrictions. Having two people (or more) is recommended for collecting samples efficiently and safely.

A challenge of this type of wastewater sampling is the quick turnaround time that is needed to produce actionable information. For this reason, the logistics of doing all sampling and processing in 1 day was often strenuous and tiring. Late nights in the lab posed potential safety problems for the person processing the samples. With practice, this process was slowly streamlined to take less time, but in future projects, it is recommended to have multiple people working on sampling and processing to reduce deadtime and any bottlenecks. Other ideas for reducing overall time would be to have more teams sampling to be able to return the samples to the lab more quickly. Our lab originally only had one centrifuge which resulted in a lack of space for all samples at once. Although we eventually acquired a second, more centrifuges would have been useful in making the process more efficient. Adequate resources need to be allocated to permit timely collection and analysis of samples and allow prompt action in the event of a positive sample. A goal would be to have samples collected on a given day and be analyzed overnight and available the next morning.

One additional factor to consider is that of dilution. Unrelated wastewater analysis, also testing for the presence of SARS‐CoV‐2, noted a high variability within samples because of flow dilution. This led to a process known as normalization, which adjusts a wastewater sample relative to the fecal matter present. However, although this would be beneficial to help ascertain the “true” amount of SARS‐CoV‐2 in a sample and therefore improve the significance of positive results, it adds additional processing time. It would therefore have to be determined if it is worthwhile to improve accuracy at the expense of a potentially delayed response. This decision may also be contingent upon the potential infectiousness and severity of a particular strain.

## CONCLUSIONS AND LESSONS LEARNED

### Summary of lessons learned

In the future, key things to consider are choosing appropriate sites, gathering and storing materials needed, and planning for weather challenges. It is important to develop an iterative process that works for the particular campus being monitored. When establishing a plan to implement a similar wastewater surveillance study on a large campus, it is also crucial to note all of the following:

- Sewer maps may be outdated and not frequently updated; clarifications may not be available.
- Many sewers catch sewage from multiple buildings, thus making it difficult to relate the results to one location.
- Agreements about which locations a site is drawing from must be clarified prior to an initiative.
- Many access points can be located on roads or high‐trafficked areas and thus must be avoided.
- Autosampler batteries need to be frequently recharged; more often in cold weather. Sufficient supplemental batteries should are required to accommodate this factor.
- Strainers in the sewers occasionally needed to be mounted or readjusted in order to keep the tubing for sampling below the water level in the sewer.
- Toilet paper and wipes can clog the strainer and limit the sample collection.
- Ensure all parts are working on the autosamplers and have a contingency plan in a device or part of a device fails.
- If possible, the tubing lines in the sampler should be purged and the sampling ends withdrawn over winter or an extended break so as to avoid complications that may arise from sitting sewage.
- Specific insulation or tubing may be required to further prevent ice build‐up.
- Multiple teams collecting samples can reduce the amount of time from when a sample is collected to when a sample is processed.
- Ensure enough laboratory equipment (e.g., centrifuges) is available to meet all sampling goals.
- Map coordination and planned driving can assist in navigating sampling sites.
- Determine in advance whether a sewer has a lower flow and would thus require a low‐flow strainer.
- Have a proper method to insulate and heat, if needed, the tubing to help prevent obstacles created by low temperatures.

### Conclusions and recommendations for future work

Based on the experiences of this project, wastewater monitoring is a doable process that can be implemented across colleges or other campuses to detect SARS‐CoV‐2. The logistical infrastructure is present to survey a large population of people to obtain a representative sample for early detection and quick response time. However, careful planning and contingencies are essential for a successful implementation. As SARS‐CoV‐2 mutates, it will have to be seen if WBE will still remain a highly effective tool for campuses, communities, etc. However, to date, WBE has been successful in the detection of many variants (Hrudey & Conant, 2022), including Alpha, Beta, Gamma, Delta, Lambda, Mu, and Omicron (Hrudey & Conant, 2022; Kirby et al., 2022; Oloye et al., 2022; Sutton et al., 2022; Wolfe et al., 2022). Additionally, this type of wastewater surveillance program may be useful in other ways, namely, in detecting other pathogens and viruses in wastewater such as *E. coli* or *salmonella*.

## CONFLICT INTEREST

The authors declare that they have no known competing financial interests or personal relationships that could influence the work and results presented in this paper.

## Supporting information


**Data S1.** Supporting Information

## Data Availability

The data that support the findings of this study are available upon request to University of Maryland.
