# Supplementary material for: Implementing wastewater surveillance for SARS‐CoV‐2 on a university campus: Lessons learned
Source: Water Environ Res. 2022 Nov 13;94(11):e10807. doi: 10.1002/wer.10807 (PMC9827968; doi:10.1002/wer.10807)
Supplement: Supplementary file 1 — Data S1. Supporting Information [file WER-94-0-s001.docx]

**SUPPLEMENTARY INFORMATION DOCUMENT**

1. **Viral detection methods and laboratory procedures**

*Sample concentration and quantification*

Wastewater samples were collected from each site in 500 mL or 1000 mL containers. After sufficient shaking/mixing, 90 mL of each sample were poured, as two 45 mL aliquots. These aliquots were then concentrated to approximately 1 mL using the following method:

**Centrifugation:**

Each 45 mL aliquot, in 50 mL Falcon^®^ conical tubes, were centrifuged in a benchtop centrifuge (Allegra X-22, Beckman-Coulter, Brea, CA, USA) with a swinging-bucket rotor, at a speed of 3,400 G for 20 min to remove the solid fraction and large particles. The resulting supernatant was transferred to new 50 mL Falcon centrifuge tubes and apportioned over three centrifugal cycles into 15 mL Amicon Ultra-15 Centrifugal Filter Devices with a cut-off of 100 kDa (Millipore, Amsterdam, the Netherlands) for 15 min at 3,400 G. If the liquid did not pass through the device during the allotted runtime, additional centrifugation time was provided.

**Concentration:**

Once centrifugation yielded a total residual liquid (i.e., two 45 mL aliquots combined) of less than 1.5 mL, the duplicate samples were combined, and the concentrated sample was adjusted to 1 mL with RNase-free water. After rinsing and agitation, the sample was transferred to sterile 1.5 mL RNase-free Microfuge tubes (Invitrogen, Carlsbad, CA, USA) for subsequent RNA extraction.

**RNA Extraction:**

Quick-RNA^TM^ Miniprep kits (Zymo Research, Irvine, CA, USA) were used to extract RNA. Specific modification included utilizing 0.3 mL of each sample and 0.6 mL of Lysis buffer. As per the protocol, final RNA volume for each sample was 0.1 mL. Reverse transcription was performed as outlined in the publication by Kaya et al. (2022), section 2.8. Specifically, Sample RNA was converted to cDNA according to the methods outlined in the NEB #M0368 Standard Protocol (New England Biolabs, 2020). cDNA was synthesized either as 25 uL or 50 uL portions, depending upon the amount of future analysis needed.

***Table A.1.*** *Modified Standard Protocol for cDNA Synthesis*

| **Reaction mix 1:** | **vol. /25 µL, µL** | **vol. /50 µL, µL** |
| --- | --- | --- |
| Random Hexamer (primer mix) (60 µM) | 1.5 | 3 |
| dNTP mix (10mM) | 1.25 | 2.5 |
| H2O (RNase-free), µL | 3.25 | 6.5 |
| Sample RNA | 6 | 12 |
| **Total vol** | **12** | **24** |
|  | | |
| Reaction mix 1 denatured for 5 minutes at 70°C. Spun/centrifuged briefly and put on ice for 2 min. | | |
| **Reaction mix 2:** | **vol. /25 µL, µL** | **vol. /50 µL, µL** |
| 5X ProtoScript II Buffer | 5.25* | 10 |
| RNase Inhibitor (40 U/µl) | 0.5 | 1 |
| 0.1M DTT | 2.5 | 5 |
| Protoscript II RT (200U/µl) | 1* | 2.5 |
| H2O (RNase-free) | 3.75 | 7.5 |
| **Total vol** | **13** | **26** |
|  | | |
| Reaction mix 2 added and samples incubated at 42°C for 1hr. Enzyme inactivated at 70°C for 20 min and kept at 4°C until tubes removed from machine. | | |

**RT-qPCR:**

RT-PCR was conducted using a CFX Connect Real-Time PCR Detection System (Bio-Rad, Hercules, CA, USA), using standard and calibration curves to determine the viral concentration. TaqMan^®^ (Promega, Madison, WI, USA) Master primer mix was used along with specific primers and probes (Table 2). Both the N1 and N2 primer mixes were diluted to preserve stock, and it was confirmed that there was no loss, nor any significant change compared to undiluted stock. The initial viral load (RNA) in the original wastewater sample was calculated. Data was measured via RT-PCR utilizing either the N1 or N2 gene, with both genes being analyzed for comparison.

*Process Control - Bovine respiratory syncytial virus (BRSV)*

Bovine Respiratory Syncytial Virus (BRSV) (Inforce 3 Cattle Vaccine^TM^, Zoetis, Parsippany, NJ, USA) was used as a surrogate (process control), as it was used to determine the level of recovery. BRSV was chosen due to its inherent stability (Bivins et al., 2021b) and availability. Additionally, BRSV was found to functional well as a surrogate due to its morphological similarities to the SARS-CoV-2 virus and its documented use as an extraction control for drinking and wastewater samples (Bivins et al., 2021a; Bivins et al., 2021b; Valarcher and Taylor, 2007)

For each 45 mL aliquot of sample, a 1:1000 ratio (*45 µL:45 mL*) of BRSV stock concentration (median concentration of 10^11^ ± 10^1^ gc/L) was added prior to sample processing. The reported recovery determined via BRSV analysis was approximately 8.5% and most values ranged between 0.5-20% consistent with findings in similar studies (Alygizakis et al., 2020; Bivins et al., 2021b; Prado et al., 2021), Several studies (Li et al., 2021; Maestre et al., 2021) reported difficulty in utilizing BRSV as a normalization approach and obtaining high recoveries. However, in this study it was a beneficial surrogate to determine if a WRRF sample was negative (i.e., below the threshold), that it was not due to a procedural error, but rather either that it was a true value or that the low value was due to an error with the N-gene primer mix or standards.

***Table A.1.*** *Primers and probes used in this study.*

| **Description** | | **Oligonucleotide Sequence (5’>3’)** | **product length, bp** | **Final Conc., nM** | Ref. |
| --- | --- | --- | --- | --- | --- |
| N1 | FWD | GAC CCC AAA ATC AGC GAA AT | 72 | 67 | (Lu et al., 2020c) |
|  | REV | TCT GGT TAC TGC CAG TTG AAT CTG |  | 67 |  |
|  | Probe | [FAM]-ACC CCG CAT TAC GTT TGG TGG ACC-[BHQ1] |  | 17 |  |
| BRSV | FWD | GCAATGCTGCAGGACTAGGTATAAT | 124 | 500 | (Boxus et al., 2005) |
|  | REV | ACACTGTAATTGATGACCCCATTCT |  | 500 |  |
|  | Probe | [HEX]-ACCAAGACT-[ZEN]-TGTATGATGCTGCCAAAGCA-[3IABkFQ] |  | 250 |  |

1. **Scheduling and Sampling Details**

*Wastewater collection – expanded information*

Wastewater (WW) samples were often collected twice a week. If the desired sample was a composite sample, during Period 1, 90-200 mL of WW liquid per half-hour was collected over a 48-96 hour period. No timed programming was performed during Period 1; instead collections were scheduled for Monday and Wednesday or Thursday, weather permitting. In Period 2, the autosamplers were programmed to collect 130 mL samples initially over a 48-72 hour period and then reduced to a 24-30 hour period. Each sampler had an internal timer that controlled the start of sampling, and the days on which samples would be collected. Once schedules were established, samples were collected on Mondays and Thursdays between 10am and 1pm, weather permitting. The samplers were programmed to start sample collection at 6 am on the previous morning (Sunday or Wednesday) and drew samples every 30 minutes. In the month of February 2021, there were difficulties in collecting samples during Thursdays, as the weather forecast consistently called for inclement weather conditions. In these cases, collection was scheduled for Wednesday afternoon, and samples were a combination of composite and grab samples. This alternative collection schedule could be modified as needed.

For sample retrieval, a team of two people would visit each collection site and fill up labeled HDPE sample bottles, which were transported back to the lab in a cooler filled with ice or ice blankets. Eye protection or splash guards were highly recommended and lab coats were worn in the absence of winter jackets. However, due to COVID-19 safety protocols, N-95 or N-94 masks were advised and were mandated during Period 2. For general safety, disposable gloves were required, and hand sanitizer was used as needed after a collection. To prevent any pathogens from being inside the cooler or in the transportation vehicle before bottles were placed in the cooler, the outside of each sample bottle, and any soiled equipment, was sanitized with ethanol (70% v/v). For ease of use, a plastic caddy was used to carry all of the necessary materials for collection. In this caddy, the sample bottles, storage box keys, sanitizer, extra gloves, and paper towels could be stored and carried.

Upon arriving at each site, the large collection jug was inspected to determine it had collected the requisite WW sample volume (>500mL). When this was true, the collection jug was removed from within the autosampler and lightly shaken to disturb the solid formation and create a more homogenous composite sample. A smaller sample was then taken from this collection jug. Sample size varied, as both 500 mL and 1,000 mL HDPE sample bottles were used. During Period 1, both sizes were used indiscriminately. During Period 2, 500 mL sample bottles were reserved for composite samples, while 1,000 mL bottles were used for grab samples. On average, the sample bottles were filled to 50-85% of their capacity.

Once a sample bottle was filled, the remaining liquid in the collection jug was carefully poured back into the sewer with the aid of a plastic funnel. In all locations save for one site, the manhole cover was left slightly propped open to allow for this liquid waste to enter, as well as to not pinch the tubing inside it. Only one manhole cover had two holes in it, thereby allowing the tubing to enter through one and the funnel to be placed in the other. Any wastewater spills were sprayed with the ethanol mixture and wiped with paper towels, as necessary. Clean tap water was used to rinse the collection jug, allowing any solids left in the jug to be washed away. Once the collection jug was rinsed, it was returned to the inside of the autosampler with the hose placed inside, the sampler was programmed to start the next collection time.

Batteries were replaced once a week, or as needed. Cold temperatures and the new autosampler model (6712) would drain the battery faster, and thus in some cases it was necessary to replace the battery twice a week. Once at the lab, the bottles were shaken and mixed thoroughly to provide a representative sample. The samples with a higher solids content were shaken more vigorously to disturb any solids that may have settled to the bottom.


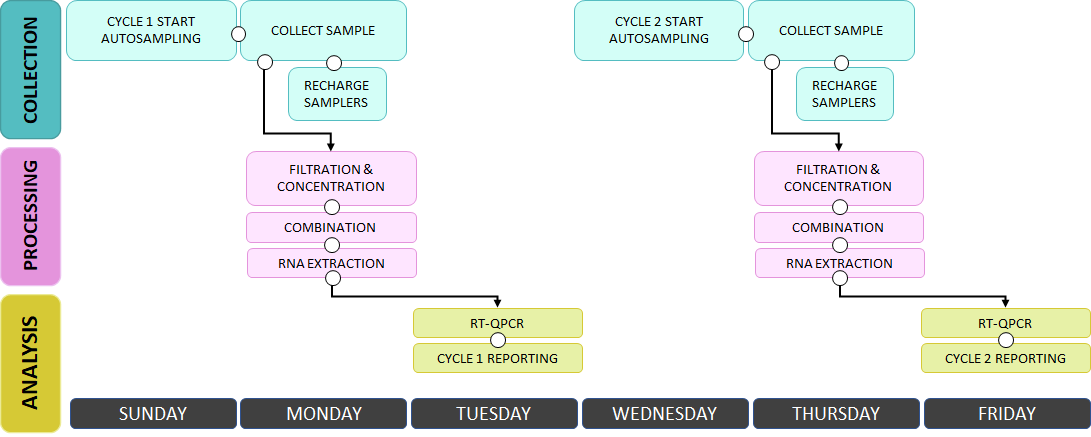


**Figure B.1.** Sample schedule for S1 and the first week of S2 (48-96 Hours).

**
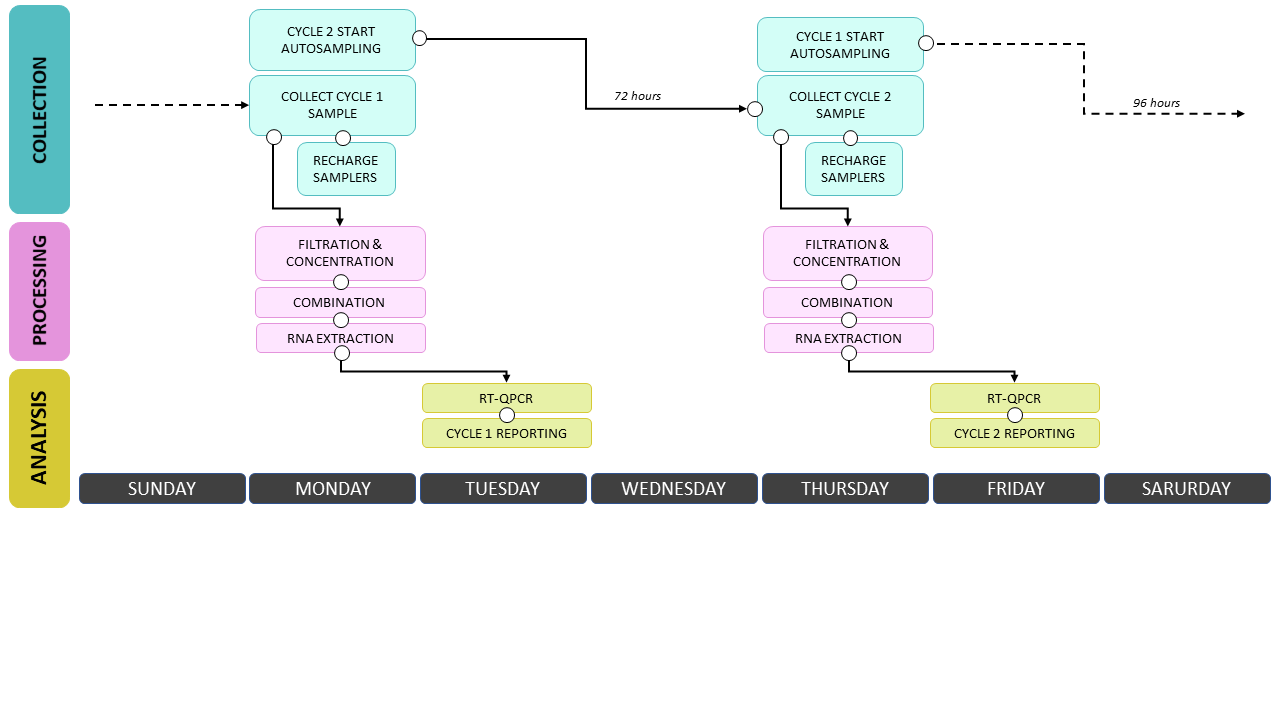
Figure B.2.** Sample schedule collection during S2 (24-30 Hours).

**
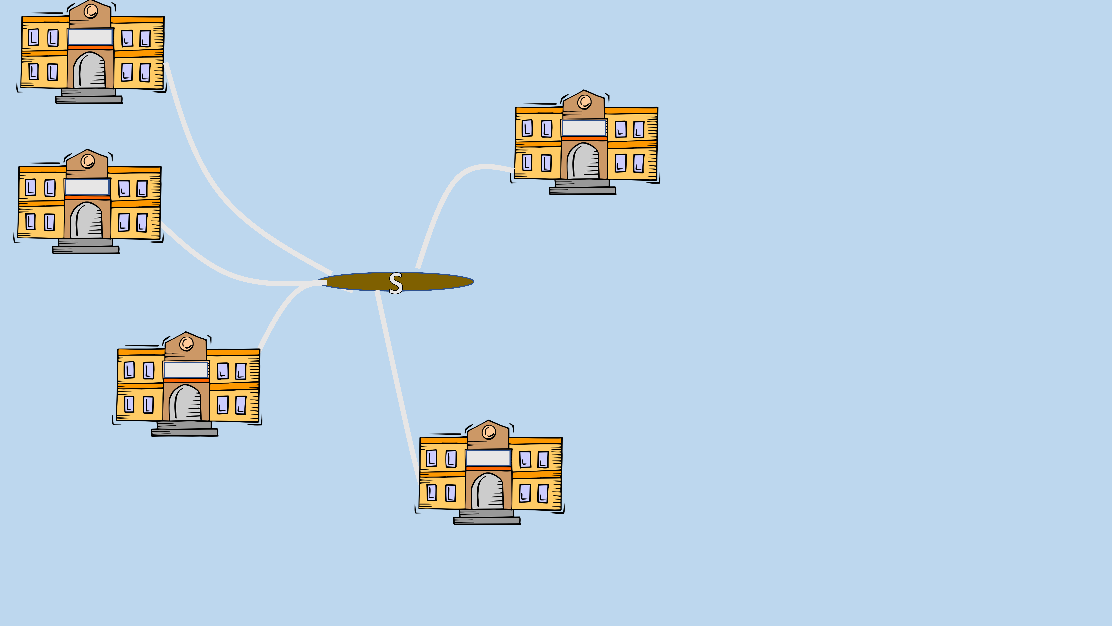

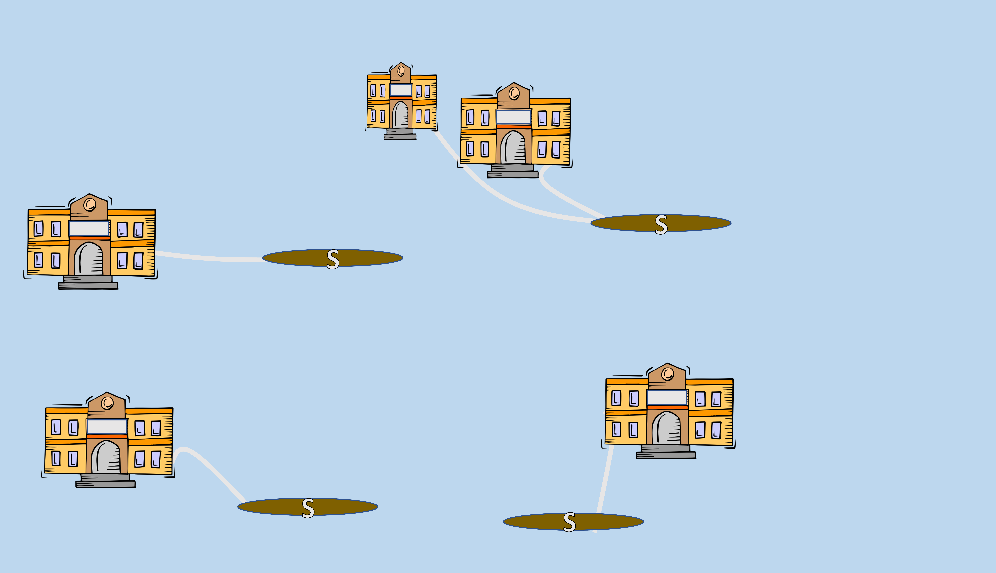
**

**Figure B.3.** Sampling patterns differences between Period 1 and Period 2. *

*The image on the left is a rough representation an example of a collection site from Period 1, pooling in from multiple dorms. The image on the right is a rough representation of the collection from Period 2.
